# Umbilical Cord Blood and iPSC-Derived Natural Killer Cells Demonstrate Key Differences in Cytotoxic Activity and KIR Profiles

**DOI:** 10.3389/fimmu.2020.561553

**Published:** 2020-10-15

**Authors:** Benjamin H. Goldenson, Huang Zhu, YunZu Michele Wang, Naveen Heragu, Davide Bernareggi, Alessa Ruiz-Cisneros, Andres Bahena, Eivind Heggernes Ask, Hanna Julie Hoel, Karl-Johan Malmberg, Dan S. Kaufman

**Affiliations:** ^1^Division of Regenerative Medicine, Department of Medicine, University of California, San Diego, San Diego, CA, United States; ^2^Department of Cancer Immunology, Institute for Cancer Research, Oslo University Hospital, Oslo, Norway; ^3^The KG Jebsen Center for Cancer Immunotherapy, Institute of Clinical Medicine, University of Oslo, Oslo, Norway; ^4^Department of Medicine Huddinge, Center for Infectious Medicine, Karolinska Institutet, Stockholm, Sweden

**Keywords:** natural killer cells, cancer, stem cells, umbilical cord blood, KIRs

## Abstract

Natural killer (NK) cells derived or isolated from different sources have been gaining in importance for cancer therapies. In this study, we evaluate and compare key characteristics between NK cells derived or isolated from umbilical cord blood, umbilical cord blood hematopoietic stem/progenitor cells, peripheral blood, and induced pluripotent stem cells (iPSCs). Specifically, we find CD56^+^ NK cells isolated and expanded directly from umbilical cord blood (UCB56) and NK cells derived from CD34^+^ hematopoietic stem/progenitors in umbilical cord blood (UCB34) differ in their expression of markers associated with differentiation including CD16, CD2, and killer Ig-like receptors (KIRs). UCB56-NK cells also displayed a more potent cytotoxicity compared to UCB34-NK cells. NK cells derived from iPSCs (iPSC-NK cells) were found to have variable KIR expression, with certain iPSC-NK cell populations expressing high levels of KIRs and others not expressing KIRs. Notably, KIR expression on UCB56 and iPSC-NK cells had limited effect on cytotoxic activity when stimulated by tumor target cells that express high levels of cognate HLA class I, suggesting that *in vitro* differentiation and expansion may override the KIR-HLA class I mediated inhibition when used across HLA barriers. Together our results give a better understanding of the cell surface receptor, transcriptional, and functional differences between NK cells present in umbilical cord blood and hematopoietic progenitor-derived NK cells which may prove important in selecting the most active NK cell populations for treatment of cancer or other therapies.

## Introduction

Natural killer (NK) cells are innate immune lymphocytes with the ability to rapidly recognize and exhibit cytotoxicity toward tumor and virus infected cells in a HLA-independent manner (1). These unique properties have led to growing use of different allogeneic NK cell adoptive transfer approaches to be used for cancer treatment (2). Sources of primary NK cells used in these clinical trials to treat both hematologic malignancies and solid tumors include peripheral blood (PB)-derived, umbilical cord blood (UCB)-derived, and induced pluripotent stem cell (iPSC)-derived NK cells (3–12). However, despite clinical use of these diverse NK cell populations, there is relatively little known about key phenotypic, genotypic, and functional comparison between these NK cell populations that may lead to difference in clinical efficacy (10, 13–16).

NK cells were first described as a lymphocyte population able to detect and rapidly kill tumor cells or viral infected cells without prior sensitization (17, 18). A key observation that NK cells could kill tumor cell lines that had lost expression of MHC class I surface molecules, and the finding that MHC class I expressing cells were resistant to lysis by NK cells, led to the formulation of the “missing self-recognition hypothesis” which states that NK cells are able to detect and kill target cells that do not express MHC class-I molecules (19, 20). The missing self-recognition hypothesis has been refined following the discovery of multiple germline encoded inhibitory and activating NK receptors (21–23). In humans, the main inhibitory receptors are inhibitory killer Ig-like receptors (KIRs), which recognize groups of HLA class-I alleles and the CD94/NKG2A heterodimer which is specific for the HLA-E molecule (24–26).

Inhibitory KIRs contain two (KIR2D) or three (KIR3D) polymorphic extracellular immunoglobulin (Ig)-like domains followed by long (L) cytoplasmic tails harboring two immunoreceptor tyrosine-based inhibition motifs (ITIMs). Four major inhibitory KIRs specific for epitopes found on distinct groups of HLA class I allotypes include KIR2DL1 which recognizes the HLA-C2 epitope, KIR2DL2/L3 which recognizes the HLA-C1 epitope, KIR3DL1 which recognizes HLA-B, or HLA-A epitopes and KIR3DL2 which recognizes HLA-A^*^03 and -A^*^11 epitopes (22, 24). NK cells also express germ-line encoded activating NK cell receptors and co-receptors that induce NK cell activation through interactions with various ligands that are expressed on tumor-transformed or virus-infected cells (27–29). Activating KIRs differ from inhibitory KIRs in that the intracellular cytoplasmic tail of activating KIRs is shorter, lacks ITIMs, and interacts with the signaling adaptor protein DAP12 that contains immunoreceptor tyrosine-based activation motifs (ITAMs) (30–32). Among the activating KIRs, KIR2DS1, and its ligand HLA C2 is the best defined receptor-ligand pair with an influence on NK cell function (33, 34).

KIRs have been shown to be clinically significant in allogeneic hemopoietic stem cell transplant (HSCT) treatments for acute myeloid leukemia (AML) in cases of KIR profile mismatches where the inhibitory KIRs on the NK cells derived from the stem cell donor do not encounter their cognate ligands on the transplant recipient's AML blasts. In these cases, NK cells become activated and kill their targets more effectively leading to a survival benefit to AML patients (35, 36). These findings have been adopted in practice, as when otherwise similarly HLA-matched allogeneic or haploidentical HSCT donors are available, HSCT donor selection algorithms consider KIR profiles as a donor selection criteria (35, 37–39). However, selection of KIR ligand mismatches in HSCT remains an active area of investigation, as a recent study found that optimization of donors based on KIR2DS1 and KIR3DL1 expression did not provide a survival benefit (40).

Expression and function of KIRs in PB-NK, UCB-NK, and iPSC-derived-NK cells have not been well-defined. UCB provides an internally genetically-controlled model to compare the CD56^+^ NK cells that are present in a cord blood unit (when collected at birth) to genetically identical NK cells derived from cord blood CD34^+^ hematopoietic stem/progenitor cells from the same cord blood unit. We find the expression of NK cell surface antigens, activating and inhibitory receptors between the different NK cell types are relatively similar, but with variable KIR expression linked to expression of markers for NK cell differentiation. To better understand the functional importance of this difference in KIR expression, we analyzed the cytotoxic function of the cells against targets expressing or lacking specific KIR ligands and demonstrate that the NK cells different KIR profiles are a significant mediator of the difference in the cells killing function.

## Materials and Methods

### Derivation and Expansion of NK Cells From Umbilical Cord Blood and Peripheral Blood

Umbilical cord blood units were purchased from the San Diego Blood Bank. Donor consent was obtained and the study followed guidelines of the UCSD Institutional Review Board. UCB56 NK cells were isolated directly from each unit using CD56 positive selection beads (Miltenyi Biotec, Bergisch Gladbach, Germany). The UCB56 NK cells were then expanded under the culture conditions described previously and cryopreserved. UCB56 cells were then thawed prior to use (41). CD34 expressing hematopoietic stem cells were also isolated from the same units using CD34 positive selection beads (Miltenyi). Using previously published protocols, the CD34+ cells were differentiated into UCB34 NK cells from each unit (10, 42). In brief, CD34+ cells were plated on irradiated OP9-DLL4 cells in medium containing a 2:1 mixture of Dulbecco modified Eagle medium/Ham F12 (Thermo Fisher Scientific, Waltham, MA, 11965092, 11765054), 2 mM L-glutamine (Thermo Fisher Scientific, Waltham, MA, 25030081), 1% penicillin/streptomycin (Thermo Fisher Scientific, Waltham, MA, 15140122), 25 μM β-mercaptoethanol (Thermo Fisher Scientific, Waltham, MA, 21985023), 20% heat-inactivated human serum AB (Corning, NY, U.S., MT35060CI), 5 ng/mL sodium selenite (Merck Millipore, Burlington, MA, S5261), 50 μM ethanolamine (MP Biomedicals, ICN19384590), 20 mg/mL ascorbic acid (Merck Millipore, Burlington, MA, A4544), interleukin-3 (IL-3; R&D Systems Minneapolis, MN, 203-IL); for first week only), stem cell factor (SCF; R&D Systems Minneapolis, MN, 7466-SC), interleukin-15 (IL-15; R&D Systems, 247-ILB), Fms-like tyrosine kinase 3 ligand (FLT3L; R&D Systems Minneapolis, MN, 308-FK), and interleukin-7 (IL-7; R&D Systems Minneapolis, MN, 207-IL). The cells were cultured in these conditions for 28–35 days receiving weekly media changes until they developed into CD45^+^CD56^+^CD33^−^CD3^−^ cells as determined by flow cytometry (43). Peripheral blood NK cells were isolated and expanded in culture as previously described. Briefly, CD56+ NK cells were positively selected from a buffy coat prepared from peripheral blood obtained from the San Diego Blood Bank by Ficoll gradient centrifugation (Miltenyi). PB-NK were then co-cultured with irradiated K562 cells in NK cell media (41).

### HLA and KIR Genotyping

HLA and KIR genotypes were determined by the UCSD Health Clinical Laboratories. HLA and KIR Luminex-based tissue typing assays using sequence-specific oligonucleotides (SSO) Genotyping Kits were used (One Lambda, Thermo Fisher). HLA typing was performed by direct DNA sequence analysis using next-generation DNA sequencing technology in conjunction with allele sequence-specific primer PCR. DNA sequence data was analyzed by alignment and comparison with IMGT database of known HLA DNA sequences. Allele types unresolvable with available reagents were assigned NMDP G group codes. KIR ligands were assigned based on the amino acid residues at position 77 and 80 of each HLA B and HLA C protein (Bw4 with N/D/S at residue 77 and I/T at residue 80; Bw6 with G/S at residue 77 and N at residue 80; C1 with N at residue 80; and C2 with K at residue 80) (44, 45). For HLA serological typing, serological equivalents were derived from the molecular typing results.

### Cell Lines and Cell Culture

Neuroblastoma cell lines SK-N-AS, IMR32, and NBLS were provided by Dr. Peter Zage (UCSD Moores Cancer Center), and their identities were validated by American Type Culture Collection (ATCC, Manassas, Virginia, U.S.). The 721.221 lymphoblastic cell line (221.wt) that was transfected with HLA cw3 (C1) and HLA cw4 (C2) was generously provided by Peter Parham. K562, NK-92, Jurkat, and Raji cell lines were obtained from ATCC. Raji, K562, 721.221.wt, and neuroblastoma lines were cultured in RPMI 1640 media (Thermo Fisher Scientific, Waltham, MA, 11875085) supplemented with 2 mM glutamine (Sigma) and with 10% heat-inactivated fetal bovine serum (FBS) (Sigma). 221.C1/C2 lines were maintained with Geneticin (Thermo Fisher Scientific). NK-92, K562, Raji and Jurkat cells were sub-cultured following ATCC recommendations.

### Derivation and Expansion of NK Cells From iPSCs

The derivation of NK cells from iPSCs has been previously described (9, 11, 43). Briefly, 8,000 TrypLE-adapted iPSCs were seeded in 96-well round-bottom plates with APEL2 media (Stem Cell Technologies, Vancouver, BC, Canada) containing 40 ng/ml human Stem Cell Factor (SCF), 20 ng/ml human Vascular Endothelial Growth Factor (VEGF), and 20 ng/ml recombinant human Bone Morphogenetic Protein 4 (BMP-4). After day 8 of hematopoietic differentiation, spin embryoid bodies were then directly transferred into each well of uncoated 24-well plates under a condition of NK cell culture. Cells were then further differentiated into NK cells using 5 ng/mL IL-3 (first week only), 10 ng/mL IL-15, 20 ng/mL IL-7, 20 ng/mL SCF, and 10 ng/mL flt3 ligand for 28–32 days. iPSC-NK cells were expanded using irradiated K562-IL21-41BBL cells, also termed artificial antigen presenting cells (aAPCs) (41).

### Flow Cytometry

Flow cytometry was done on a BD FACS Calibur, BD LSRII or NovoCyte 3000, and data were analyzed using FlowJo or NovoExpress.

### Antibodies

The following antibodies were used for flow cytometry (all anti-human): CD16-PE (BD Biosciences, 560995, clone 3G8), CD16-APC (BD Biosciences, 561248, clone 3G8) NKG2D-PE (BD Biosciences, 557940, clone 1D11), NKp44-PE (BD Biosciences, 558563, clone p44-8), NKp46-PE (BD Biosciences, 557991, clone 9E2), TRAIL-PE (BD Biosciences, 565499, clone YM366), FAS ligand-PE (BD Biosciences, 56426, clone NOK-1), NKG2A-PE (Beckman Coulter, IM3291U, clone Z199), CD158a,h (KIR2DL1, KIR2DS1)-PE (Beckman Coulter, A09778, clone EB6B), CD158b1/b2,j (KIR2DL2, KIR2DL3, KIR2DS2)-PE (Beckman Coulter, IM2278U, clone GL183), CD158e1(KIR3DL1)-BV421 (BioLegend, 312713, clone DX9), CD158a(KIR2DL1)-APC (Miltenyi, 130-120-584, clone REA284), CD158b1/b2,j (KIR2DL2, KIR2DL3, KIR2DS2)-PE-Cy5.5 (Beckman Coulter, A66900, clone GL183), CD158a,h(KIR2DL, KIR2DS1)-PE-Cy7 (Beckman Coulter, A66899, clone EB6B), CD158b2(KIR2DL3)-PE, (R&D systems, FAB2014P, clone 180701), CD155-PE (BioLegend, 337609, Clone SKII.4), HLA E-PE (BioLegend, 342603, Clone 3D12) MICA/MICB-PE (BD Biosciences, 558352, Clone 6D4), CD112-PE (BD Biosciences, 551057, Clone R2.525), ULBP2/5/6-PE (R&D Systems, FAB1298P, Clone 16590).

### Mass Cytometry

**Figure 1:**
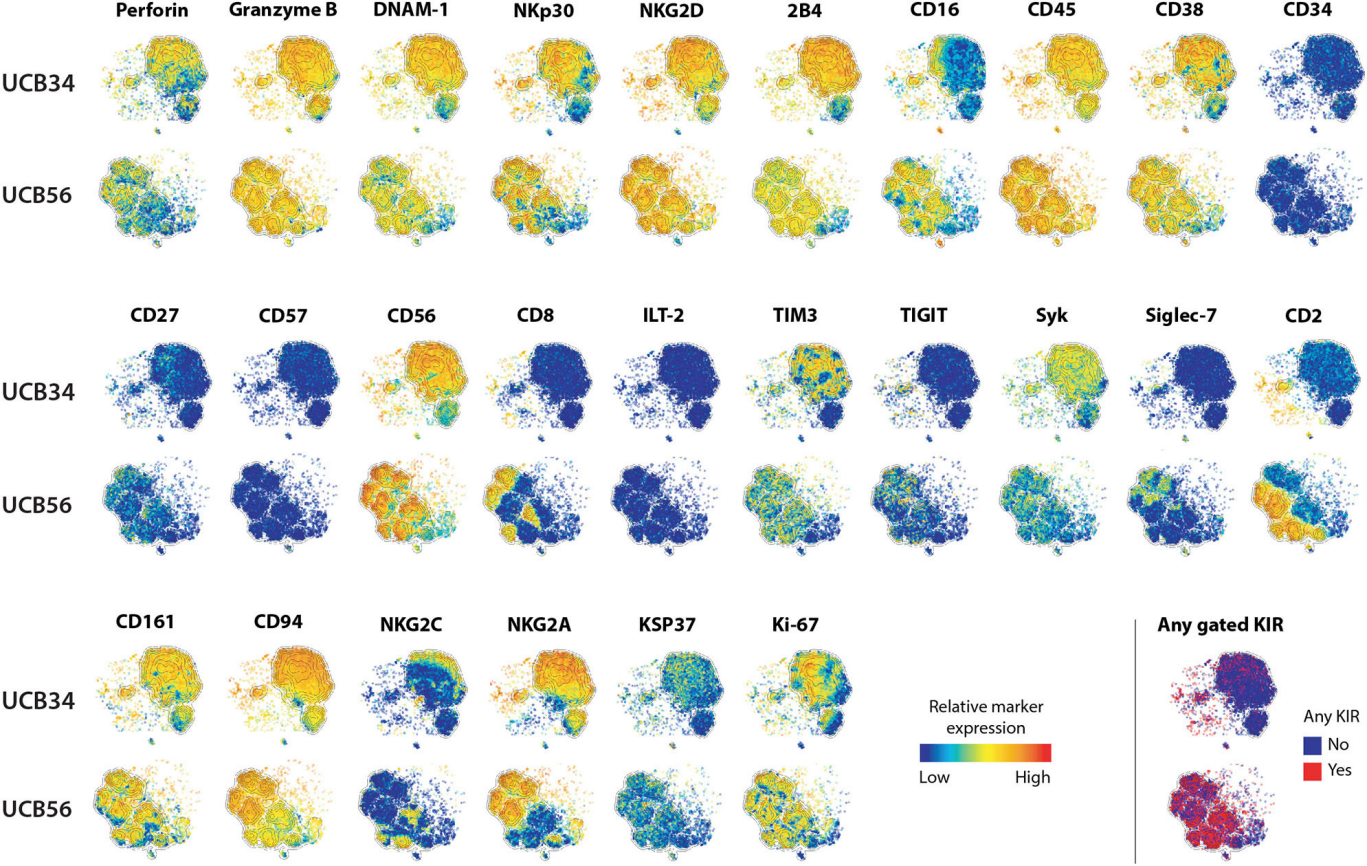
UCB56 and UCB34 NK cell phenotypes by mass cytometry. tSNE plots demonstrating the range (positive—red, negative—blue) of expression of NK cell markers of function, activity, maturation, and identity by mass cytometry. UCB34 NK cells from Donor 2 are grouped in the top rows while UCB56 NK cells from Donor 2 for each marker are grouped in the bottom rows.

**Figure 2:**
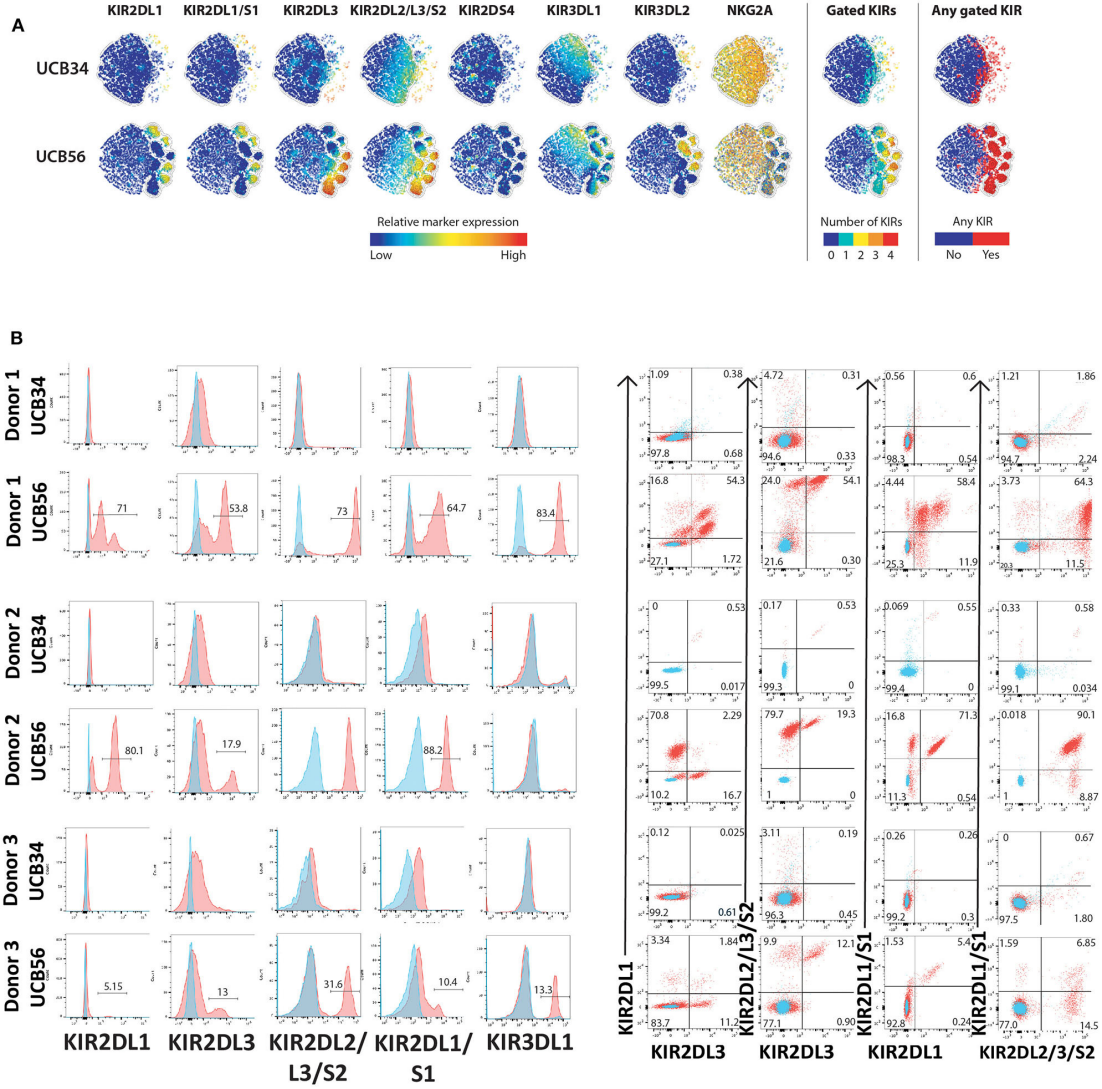
KIR Expression of UCB56 and UCB34 NK cells **(A)** KIR phenotypes by mass cytometry. tSNE plots demonstrating the range (positive—red, negative—blue) of expression of KIR markers. UCB34 NK cells from Donor 2 are grouped in the top rows while UCB56 NK cells from Donor 2 for each marker are grouped in the bottom rows. **(B)** NK cell KIR markers by flow cytometry of Donor 1, 2, and 3 UCB34 and UCB56 NK cells (red) compared to isotype controls (blue). Representative panels are shown from *n* = 3 replicates.

For viability assessment, cells were stained with Cell-ID Intercalator-103Rh (Fluidigm, San Francisco, CA, 201103B) in complete medium for 20 min at 37°C. Maxpar Cell Staining Buffer (Fluidigm, 201068) was used for all antibody staining and subsequent washing. Samples were incubated with Fc receptor binding inhibitor (Thermo Fisher Scientific, 14-9161-73) for 10 min at room temperature, before adding surface antibodies and incubating for 30 min at 4°C. Subsequently, cells were fixed in Maxpar PBS (Fluidigm, 201058) with 2% formaldehyde, transferred to methanol and stored at −20°C. The day after, cells were stained with an intracellular antibody cocktail for 40 min at 4°C and labeled with Cell-ID Intercalator-Ir (Fluidigm, 201192B). Samples were supplemented with EQ Four Element Calibration Beads (Fluidigm, 201078) and acquired on a CyTOF 2 (Fluidigm) equipped with a SuperSampler (Victorian Airship, Alamo, CA) at an event rate of <500/s. Antibodies were either obtained pre-labeled from Fluidigm or conjugated with metal isotopes using Maxpar X8 antibody labeling kits (Fluidigm) (Supplementary Table 1). FCS files were normalized using Helios software (Fluidigm) and gated on CD45+ CD19- CD14- CD32- CD3- viable single cells using Cytobank (Cytobank Inc., Santa Clara, CA). For subsequent analysis, data was imported into R (R Core Team, 2019) using the *flowCore* package, and transformed using *arcsinh(x/5)*. Ten thousand events were randomly sampled from each file and concatenated. t-Distributed Stochastic Neighbor Embedding (t-SNE) was then performed using the *Rtsne* R package with default settings and results were visualized using the *ggplot2* R package. The following markers were used for the clustering shown in Figure 1: 2B4, CD2, CD8, CD16, CD161, CD27, CD34, CD38, CD45, CD56, CD57, CD94, DNAM-1, Granzyme B, ILT-2, Ki-67, KSP37, NKG2A, NKG2C, NKG2D, NKp30, Perforin, Siglec-7, SYK, TIGIT, and TIM-3. The clustering in Figure 2 was based on the following markers: KIR2DL1, KIR2DL1/S1, KIR2DL3, KIR2DL2/L3/S2, KIR2DS4, KIR3DL1, and KIR3DL2. t-SNE plots showing the number of expressed KIRs per cell were created by manually gating on positive cells in 5 KIR stains (KIR2DL1/S1, KIR2DL2/L3/S2, KIR2DS4, KIR3DL1, KIR3DL2) and determining for how many of these gates each cell is positive. t-SNE plots showing “any gated KIR” indicate which cells express at least one KIR, according to the described manual gating.

### Cytotoxicity Assays

#### CellEvent™ Caspase-3/7 Green Flow Cytometry Assay

Target cells were pre-stained with CellTrace™ Violet (Thermo-Fisher Scientific, C34557) at a final concentration of 5 μM in PBS for 15 min at 37°C. After staining, the cells were washed in complete culture medium prior to being mixed with NK cell cultures at the indicated effector to target (E:T) ratios. After a brief centrifugation, co-cultures were incubated at 37°C for 3.5 h. Afterwards, CellEventR Caspase-3/7 Green Detection Reagent (Thermal Fisher Scientific, C10423) was added for an additional 30 min of culture for a total incubation time of 4 h. During the final 5 min of staining, SYTOX™ AADvanced™ dead cell stain solution (Thermal Fisher Scientific, S10349) was added and mixed gently. Cells were then analyzed by flow cytometry.

#### IncuCyte Caspase-3/7 Green Apoptosis Assay

Target cells were labeled with CellTraceTM Far Red (ThermoFisher, C34564). Adherent target cells were seeded in a 96-well plate at a density of 4,000 cells/well 24 h before addition of IncuCyte Caspase-3/7 Green Apoptosis Assay Reagent (Essen Bioscience, 4440) to each well-diluted by a factor of 1,000. Non-adherent target cells were seeded in fibronectin coated 96-well plates at a density of 30,000 to 50,000 cells/well and further incubated at room temperature for 30 min before the addition of IncuCyte Caspase-3/7 Green Apoptosis Assay Reagent. After incubation, NK cells were added at various E:T ratios and monitored on the IncuCyte ZOOM to acquire images every 1 h for adherent cells and every 30 min for non-adherent cells. Experiments were performed with 3 independent biological triplicates. The cytotoxicity of target cells was analyzed by quantifying red cell number and/or overlay of Caspase 3/7 (green) within the red cells.

### RNA Sequencing and Quantitative Real Time PCR

Total RNA was isolated from cells using the RNeasy mini Kit (Qiagen, 74104) according to the manufacturer's protocol. RNA quality control, library construction, sequencing and data analysis were performed by Novogene Inc. Briefly, RNA quantification and qualification were performed using Nanodrop for checking RNA purity (OD260/OD280), agarose gel electrophoresis and Agilent 2100 for checking RNA integrity. mRNA was purified from total RNA using poly-T oligo attached magnetic beads. A cDNA library was synthesized using random hexamer primer and MMuLV Reverse Transcriptase and sequencing using Illumina Hiseq 2000. Colors descending from red to blue in heatmap of differential expression genes indicated log10 (FPKM+1) from largest to smallest. ClusterProfiler software was used for all enrichment analysis, including Gene Ontology (GO) enrichment, Disease Ontology (DO) enrichment, and Kyoto Encyclopedia of Genes and Genomes (KEGG, http:www.kegg.jp/). p_adj_ < 0.05 was considered as significant enrichment. RNAseq data was deposited at Gene Expression Omnibus (https://www.ncbi.nlm.nih.gov/geo/) with reference numbers GSE150806 and GSE150363.

For qRT-PCR complementary DNA (cDNA) was reverse transcribed from RNA isolated as above using the iScript gDNA clear cDNA Synthesis Kit (Bio-Rad, 1725034) according to the manufacturer's instructions. cDNA was used as a template in qPCR experiments using specific primers (500 nM) and Sso Advanced Universal SYBR Green Super Mix as the detection chemistry (Bio-Rad, 1725270). The thermal profile included one phase at 95°C for 3 min, second phase of 40 cycles at 95°C for 15 s, 60°C for 30 s. Melt curve analysis was run after every experiment. The experiments were carried out and analyzed with Bio-Rad CFX 96 and software (Bio-Rad). Cycle threshold (Ct) values were determined and relative mRNA contents were inferred from normalization of the gene of interest expression to that of the housekeeping gene GAPDH (ΔCt). Relative expression results were plotted as (2^−ΔCt^). Forward and reverse primer sequences are: SOS1-F: CAAATCATGGGCAGCCAAGA, SOS1-R: TCTCTTCAGCTGACTTGGCA, PRF1-F: ACCAGGACCAGTACAGCTTC, PRF1-R: GGGTGCCGTAGTTGGAGATA, GZMB-F: CTTCAGGGGAGATCATCGGG, GZMB-R: TCG TCTCGTATCAGGAAGCC, IFNG-F: TGAATGTCCAACGCAAAGCA, IFNG-R: TACTGGGAT GCTCTTCGACC, CSF2-F: GCGTCTCCTGAACCTGAGTA, CSF2-R: CAGTGCTGCTTGTAG TGGC, SH2D1A-F: CAGTGGCTGTGTATCATGGC, SH2D1A-R: TCAGCACTCCAAGAACCT GT, GAPDH-F: GTCTCCTCTGACTTCAACAGCG, GAPDH-R: ACCACCCTGTTGCTGTAG CCAA.

### Statistics

Unpaired student's *t*-test and ANOVA were used to quantify statistical deviation between experimental groups. Asterisks denote significant differences ^*^P < 0.05; ^**^P < 0.01; ^***^P < 0.001 for comparisons as indicated in figure legends.

## Results

### NK Cell Surface Antigen Expression on UCB34 NK Cells and UCB56 NK Cells

To investigate the differences between NK cells derived *in vitro* from HSCs and NK cells that develop *in utero* while controlling for genetic heterogeneity between individuals, we isolated CD56^+^ UCB NK cells (termed UCB56 NK) and CD34^+^ UCB hematopoietic stem/progenitor cells from the same donors. The UCB34 cells were then differentiated into NK cells using standard methods (termed UCB34 NK) (10, 42) (Supplementary Figure 1A). UCB34 NK cells and UCB56 NK cells were derived from 3 separate UCB units (labeled donors 1–3) and NK cells were first analyzed by mass cytometry for 37 cell surface antigens (Figure 1, Supplementary Table 1). Both the UCB34 and UCB56 cell populations expressed similar levels of NK cell activating receptors, such as NKp30 (Figure 1). Many other similarities in cell surface receptors were present between the UCB34 and UCB56 NK cells including comparable expression of markers of NK cell activity and activation including Granzyme B, Perforin, and NKG2D (Figure 1). Some key differences are notable such as UCB56 NK cells expressing higher levels of CD16, the FcgRIII receptor important to mediate NK cell antibody mediated cellular cytotoxicity (6, 15, 46) and CD2, an adhesion molecule that is tightly linked to terminal NK cell differentiation (47). These results were also investigated by flow cytometry on UCB34 and UCB56 cells from donors 1–3 (Supplementary Figure 2). Overall results were similar between flow cytometry and mass cytometry, with low NKG2A expression by flow cytometry an exception. NKp44 expression, a marker of activation, was positive in all NK cell populations examined.

### UCB34-NK Cells Lack KIR Expression

The most striking phenotypic difference between the UCB56 NK cells and UCB34 NK cells was that UCB34 NK cells had very low expression of KIRs, measured by pan-KIR antibodies (Figure 1). Expression of individual KIRs on UCB56 NK and UCB34 NK cell populations was further interrogated by mass cytometry and flow cytometry (Figures 2A,B). UCB56 NK cells from all of donors 1–3 expressed KIR2DL3 and donor 1 and 2 expressed KIR2DL1 while the UCB34 NK cells from these same donors demonstrated minimal expression of individual KIRs by mass cytometry and flow cytometry (Figures 2A,B, Supplementary Table 2). Apart from the common expression of KIR2DL3 and KIR2DL1 in the UCB56 cells, there were expected differences in KIR expression between the donors. For example, Donor 3 UCB56 cells express KIR3DL1, while Donor 2 UCB56 NK cells express KIR2DL3 and KIR2DL1 in addition to the shared KIRs (Figure 2B). Each donor expressed a slightly different KIR profile, but across all donors the lack of KIR expression in the UCB34 NK cells was consistent. The NKG2A/CD94 heterodimer, that belongs to the C-lectin receptor family, is another important inhibitory NK cell receptor that recognizes HLA-E (26, 30). Both UCB34 and UCB56 NK cells express similar levels of NKG2A by mass cytometry (Figure 1).

### Improved Cytotoxic Activity of UCB56 NK Cells Cytotoxic Activity Is Independent of Target Cell HLA Expression

**Figure 3:**
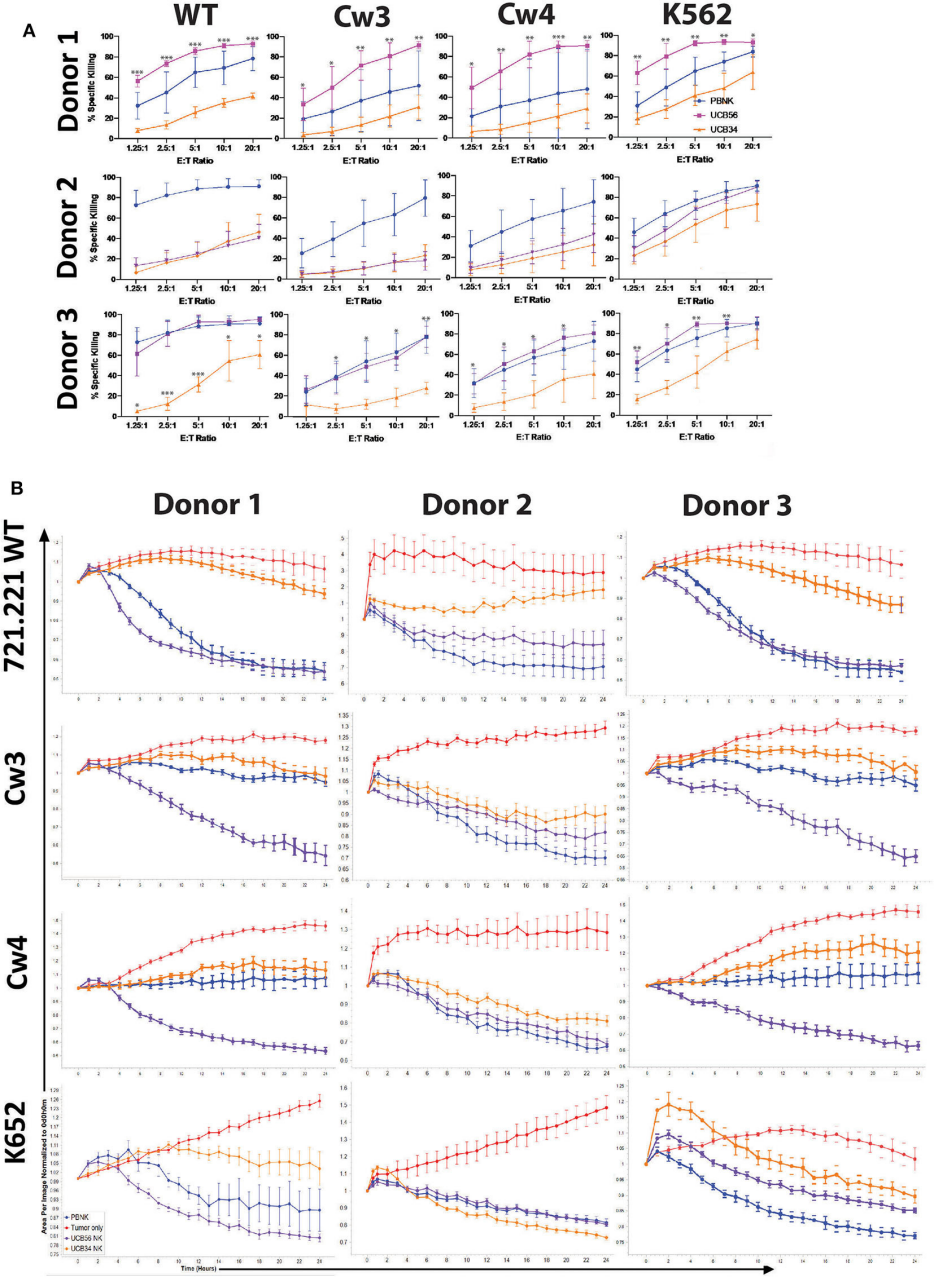
UCB56 NK and UCB34 NK cell killing activity against lymphoblastic 721.221 and myeloid K562 tumors **(A)** Cell death and apoptosis by caspase 3,7 activation, and 7-AAD staining of PBNK cells, UCB56 NK cells, and UCB34 NK cells with 721.221 WT, cw3, cw4, and K562 cells at Effector:Target ratios from 1.25:1 up to 20:1 in 4-h co-culture. Experiments were completed in triplicate and representative panels are shown from *n* = 3 replicates. All statistical analysis is of the comparisons between UCB56 and UCB34 NK cells. **(B)** Tumor cells alone (red) and tumor cell killing by PB-NK (blue), UCB56 (purple), and UCB34 NK cells (orange) measured by Incucyte live-imaging system over 24 h. Experiments were completed in triplicate. *P < 0.05; **P < 0.01; ***P < 0.001.

We next addressed whether the difference in KIR expression noted between UCB34 and UCB56 NK cells leads to differences in cytotoxic activity between the KIR^+^ UCB56 NK cells and the KIR^−^ UCB34 NK cells. To determine the functional impact of the differences noted in KIR expression, 721.221 WT lymphoblastic cells lacking HLA A, B, and C, were engineered to express HLA proteins that are KIR ligands, specifically Cw3 (HLA C1), and Cw4 (HLA C2). Class I MHC negative K562 leukemia cells were used as positive control target cells in standard cytotoxicity assays (24). KIR^+^ PB NK cells were included as a positive control effector cell population (Figure 3). Importantly, the UCB56 NK cells from each of donors 1–3 express KIR2DL3 (corresponding ligand HLA C1) and donor 1 and donor 2 UCB56NK cells also express KIR2DL1 (corresponding ligand HLA C2). Cytotoxicity assays demonstrated higher (Figure 3A, donor 1 and donor 3) or equal (Figure 3A, donor 2) activity of the KIR^+^ UCB56 NK cells compared to KIR^−^ UCB34 NK cells. This was true across all donors and for all target cell populations (Figure 3A). The addition of HLA C1 and HLA C2 expression to the 221.Cw3 and 221.Cw4 lines inhibited killing by both the UCB56 and UCB34 NK cells, though, the UCB56 NK cells maintained an improved killing ability compared to the UCB34 NK cells (Figure 3A). There were no differences in killing of the 221.Cw3 or 221.Cw4 lines, even amongst the donor 3 UCB34 and UCB56 NK cells, where the UCB56 cells expressed KIR2DL3, KIR2DL2/L3/S2, KIR2DL1/S1, and KIR3DL1 (Figure 3A). Additional long-term (24 h) cytotoxicity assays demonstrated similar or superior killing of all tumor targets by KIR^+^ UCB56 NK cells compared to the KIR^−^UCB34 NK cells (Figure 3B).

The cytotoxic activity of the KIR positive UCB56 and KIR negative UCB34 NK cells was also tested against three pediatric neuroblastoma solid tumor cell lines with different HLA haplotypes (Supplementary Figure 3A). The neuroblastoma line SK-N-AS has high expression of HLA and is HLA genotype C1/C1, the IMR32 cell line does not express HLA A, B, or C and is C1/C1, and the NBLS line has high expression of HLA and is C2/C2 (Supplementary Figure 3A). These short-term (4 h) cytotoxicity assays did not demonstrate significantly different killing by the KIR^+^ UCB56 NK cells compared to the KIR^−^ UCB34 NK cells for donors 1 and 2. Donor 3 UCB56 NK cells had significantly increased killing of each neuroblastoma cell line and the control K562 cells (Supplementary Figure 3B). Longer-term (24 h) live-imaging killing assays confirmed that KIR^+^ UCB56NK cells had equivalent cytotoxicity against the three neuroblastoma lines, consistent with the results obtained with 721.221 cells (Supplementary Figure 3C).

### iPSC-Derived NK Cell Populations That Differ in KIR Expression Show Similar Cytotoxic Activity

**Figure 4:**
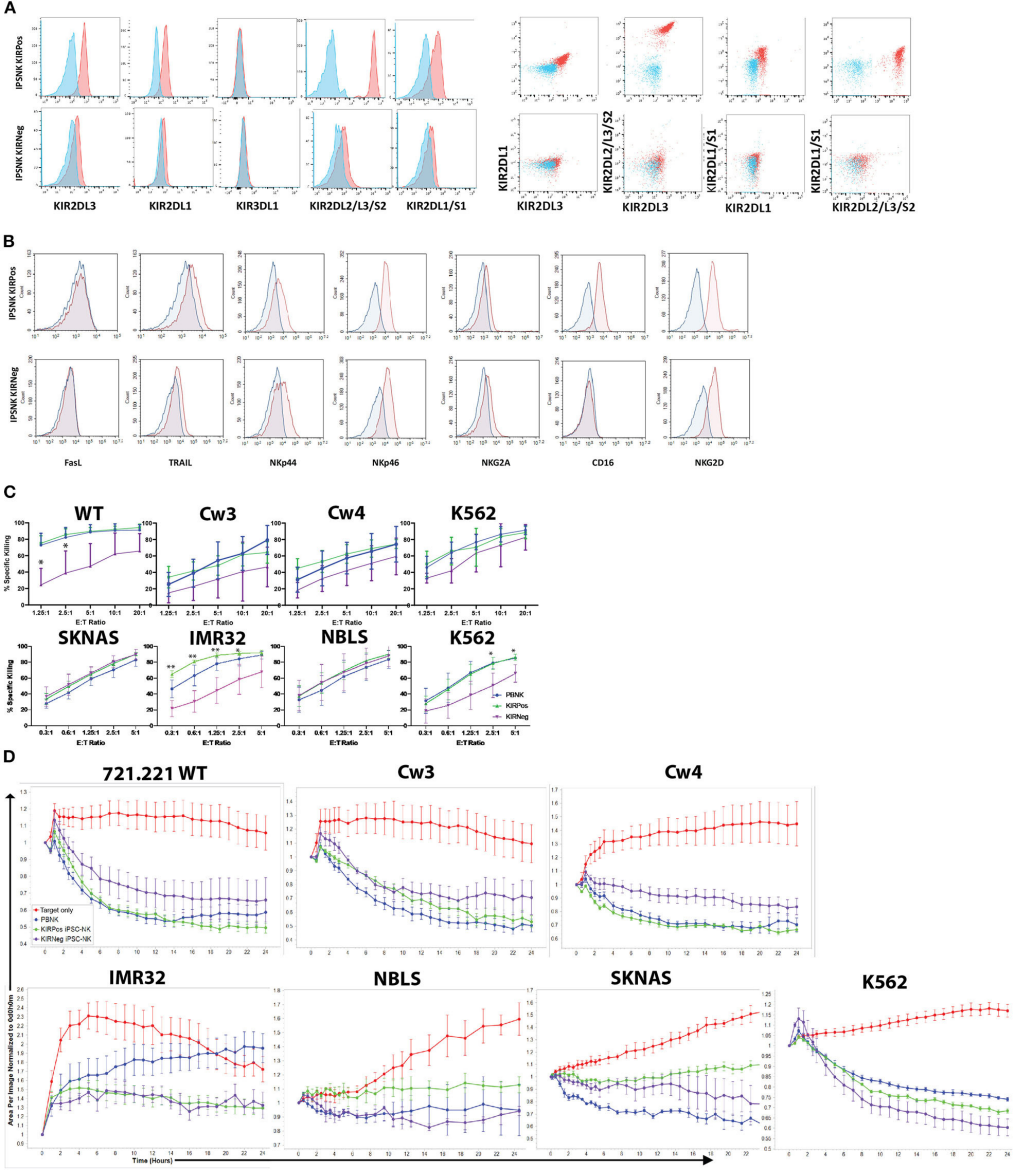
KIR expression and cytotoxic activity of iPSC-derived KIRPos and KIRNeg NK cells. **(A)** NK cell KIR markers by flow cytometry of iPSC-KIRPos and iPSC-KIRNeg NK cells (red) compared to isotype controls (blue). Representative panels are shown from *n* = 3 replicates. **(B)** NK cell markers by flow cytometry of iPSC-KIRPos and iPSC-KIRNeg NK cells (red) compared to isotype controls (blue). **(C)** Cell death and apoptosis by caspase 3,7 activation and 7-AAD staining of 721.221 WT, cw3, cw4, SK-N-AS, IMR32, NBLS, and K562 cells with PBNK cells (blue), iPSC-KIRPos NK cells (green), and iPSC-KIRNeg NK cells (purple) after 4-h co-culture at effector:target ratios from 1.25:1 up to 20:1 (721.221 cells) or 0.3:1 up to 5:1 (neuroblastoma cells). Representative panels are shown from *n* = 3 replicates. All statistical analysis is of the comparisons between KIRPos and KIRNeg iPSC-NK cells. **(D)** Tumor cells alone (red) and tumor cell killing by PBNK (blue), iPSC-KIRPos (green), and iPSC-KIRNeg NK cells (purple) measured by Incucyte live-imaging system over 24 h. Experiments are completed in triplicate. *P < 0.05; **P < 0.01; ***P < 0.001.

iPSCs are another important source of NK cells for clinical application (11, 48, 49). Given the differences that were noted in KIR expression and killing ability of UC34 and UCB56 NK cells, we extended our analysis to include iPSC-derived NK cells (Supplementary Figure 1B). We found that iPSC-derived NK cells derived from different iPSC lines demonstrated significant variability in their KIR expression (Figure 4A). iPSC-NK cells lacking KIR expression (iPSC-KIRNeg) expressed minimal KIRs while the iPSC NK cell populations expressing KIRs (iPSC-KIRPos) expressed KIR2DL3, KIR2DL1, KIR2DL2, and KIR2DL1 (Figure 4A). iPSC-KIRPos and iPSC-KIRNeg cells expressed similar levels of other activating receptors, including FasL, TRAIL, NKp44, NKp46, and NKG2D, except for CD16, which was expressed higher in iPSC-KIRPos cells (Figure 4B). In contrast to the UCB34 and UCB56 NK cells, both the iPSC-KIRPos and iPSC-KIRNeg cells did not express NKG2A (Figure 4B). Cytotoxicity assays using iPSC-KIRPos and iPSC-KIRNeg NK cells against lymphoma and neuroblastoma cell lines demonstrated that against a majority of the cell lines the iPSC-KIRNeg cells have similar killing compared to the iPSC-KIRPos NK cells (Figure 4C). Similarly, with neuroblastoma line target cells and K562 target cells in long-term (24 h) cytotoxicity assays, iPSC-KIRPos NK cells had no significantly increased killing compared to iPSC-KIRNeg NK cells (Figure 4D). Despite the difference in KIR expression, overall the iPSC-KIRPos and iPSC-KIRNeg NK cells had similar cytotoxic ability.

### Transcriptome Analysis of UCB and iPSC NK Cell Populations Show Similarity Between the NK Cells Derived From Different Sources

**Figure 5:**
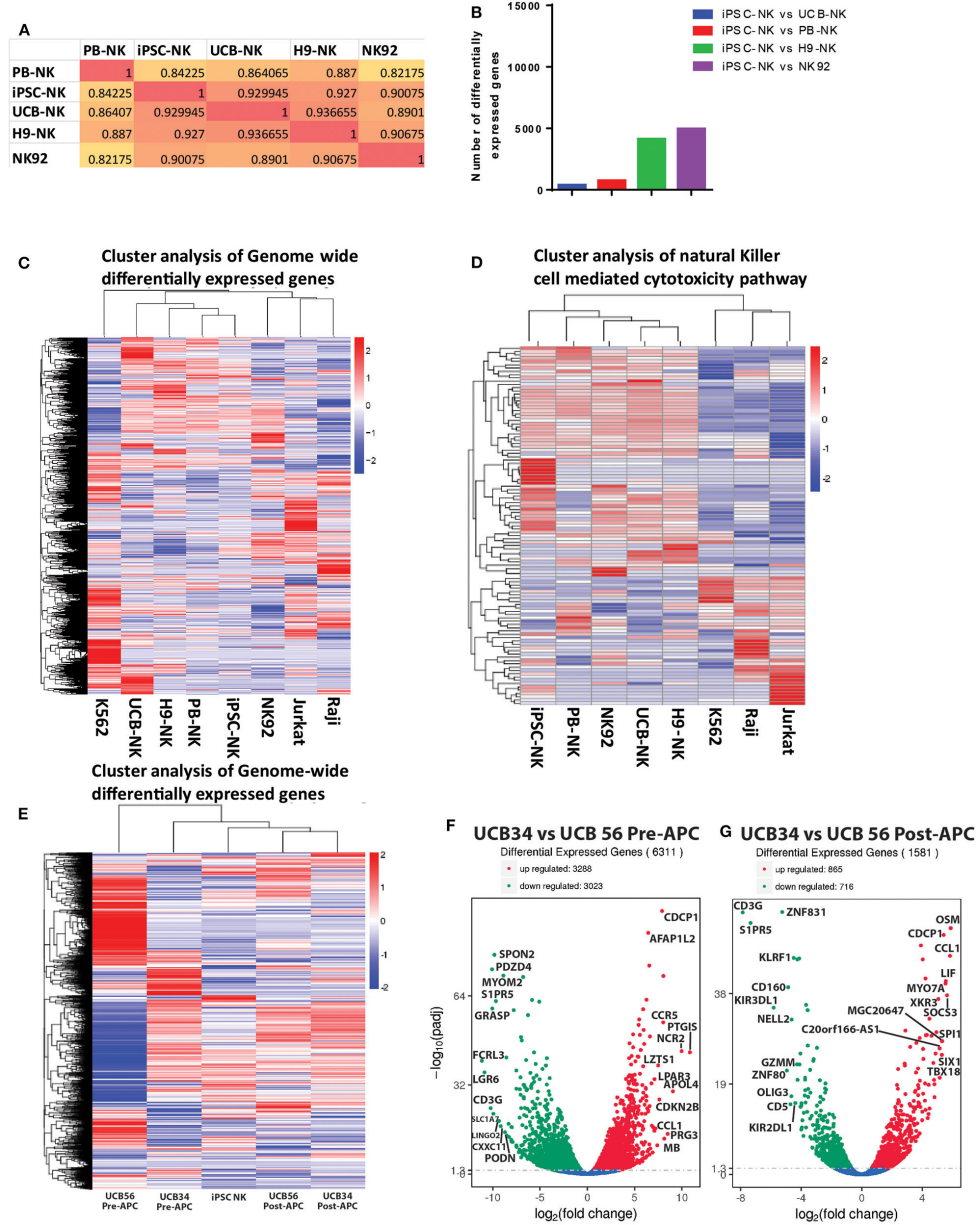
RNA Sequencing analysis of gene expression among UCB56 NK, iPSC NK, PB NK cells, NK 92 cells, and 3 cell lines, *n* = 3 biological replicates. **(A)** Heat maps of the correlation coefficient between each NK cell population. **(B)** Plot of the number of differentially expressed genes between iPSC-NK cells and the other NK cell populations. **(C)** Cluster analysis of differentially expressed genes. Genome-wide differentially expressed genes were analyzed among each cell population. Log10(FPKM+1) value was used for clustering. **(D)** Cluster analysis of genes in the NK cell mediated cytotoxicity pathway (KEGG PATHWAY: hsa04650). Hundred twenty four genes in this pathway were analyzed. **(E)** Cluster analysis of differentially expressed genes between UCB34 and UCB56 pre- and post-expansion with antigen presenting cells (APC) as well as iPSC NK cells post-expansion. Genome-wide differentially expressed genes were analyzed among each cell population. Log10(FPKM+1) value was used for clustering. **(F)** Volcano plot of differentially expressed genes between UCB34 and UCB56 cells pre-APC expansion. The x-axis indicates the fold change in gene expression between the different samples, the y-axis indicates the statistical significance (adjusted p-values) of the differences. Significantly up and down regulated genes are highlighted in red and green, respectively. Genes that were not differentially expressed between the groups are in blue. **(G)** Volcano plot of differentially expressed genes between UCB34 and UCB56 cells post-APC expansion. The x-axis indicates the fold change in gene expression between the different samples, the y-axis indicates the statistical significance (adjusted *p*-values) of the differences. Significantly up and down regulated genes are highlighted in red and green, respectively. Genes that were not differentially expressed between the groups are in blue.

Given the differences we observed between the UCB34, UCB56, and iPSC-NK cells in KIR expression and cytotoxic function, we analyzed gene expression by RNA sequencing to determine how the transcriptomes of the cell populations compared. A gene expression correlation analysis of differentially expressed genes on the whole transcriptome level between the UCB NK cells (specifically UCB56 NK cells post-APC), KIR^+^ iPSC NK cells, and PB-NK cells did not differ significantly (Figure 5A). Interestingly, when comparing the similarity of the NK cells populations by the number of differentially expressed genes to iPSC NK cells, there were the fewest differentially expressed genes in the comparison with the UCB NK cells, followed by the PB-NK cells, followed by the H9 embryonic stem cell derived-NK cells and lastly by the NK92 cell line (Figure 5B). Cluster analysis of differentially expressed genes in each NK cell population and NK-92, Jurkat, Raji, and K562 cell lines showed that the four different NK cells populations (PB-NK, UCB-NK, H9-NK, iPSC-NK) cluster together with iPSC-NK cells having the closest relation with PB-NK in terms of genome-wide gene expression (Figure 5C). In the other clusters, the lymphoid NK-92, Jurkat, and Raji cells lines group together, while the myeloid K562 cells were in a separate cluster (Figure 5C). A separate clustering analysis focused on 124 genes involved in NK cell mediated cytotoxicity pathways (KEGG PATHWAY: hsa04650) to better parse out the differences in NK cell killing activity. Here, the NK cell populations clustered together separate from the non-NK cells. The UCB NK cells clustered with the H9 NK cells, while the NK92 cells, PB-NK and iPSC NK cells clustered separately (Figure 5D). Reverse-transcription quantitative real time PCR (qRT-PCR) analysis confirmed gene expression changes seen in the RNA sequencing analysis. The separate clustering of the iPSC NK cells is defined by lower expression of signaling genes in the NK cell cytotoxicity pathway such as *SOS1* and higher expression of *SH2D1A* compared to UCB and PB NK cells. These gene expression changes were seen in both the RNA sequencing and qRT-PCR experiments (Supplementary Figure 4). Differential expression of genes in the NK cell cytotoxicity pathway such as Perforin *(PRF1)*, Granzyme B *(GZHB)*, Interferon gamma *(IFNG)*, and GM-CSF *(CSF2)* had a more variable expression pattern between the three cell types that was consistent between both the RNA sequencing and qRT-PCR analyses and was unlikely to drive the clustering (Supplementary Figure 4).

A second RNA sequencing analysis explored the transcriptional differences between the UCB34 NK cells, UCB56 NK cells and iPSC NK cells. UCB34 and UCB56 cells were analyzed both pre- and post- expansion (using irradiated aAPCs) while iPSC NK cells were expanded prior to analysis (41). Cluster analysis of differentially expressed genes in each NK cell population showed that the pre-expansion UCB34 and UCB56 cells were distinct populations (Figure 5E). However, post-expansion the UCB34 and UCB56 cells clustered together while the iPSC NK cells were more closely related to the post-APC expanded UCB cells (Figure 5E). An analysis of differentially expressed genes demonstrates a higher number of significantly differentially expressed genes between UCB34 and UCB56 NK cells pre-APC expansion with 3,288 genes differentially up-regulated and 3,023 down-regulated (Figure 5F). Post-APC expansion 865 genes were differentially up-regulated significantly between the two cell populations while 716 genes were down-regulated (Figure 5G). Notably in the post-APC expansion UCB34 cells KIR2DL1 and KIR3DL1 were expressed at significantly lower levels compared to the UCB56 cells (Figure 5G).

## Discussion

In these studies, we demonstrate that UCB56 NK cells have consistently better cytotoxic activity compared to UCB34 NK cells derived from the same donor (sharing the same KIR and HLA genotypes). Other than expression of KIRs, CD2, and CD16, the UCB34 and UCB56 NK cell populations exhibited similar cell surface receptors. At the transcriptome level, important differences identified between the UCB56 and UCB34 NK cells include differential expression of KIR transcripts, and differential expression of genes important in differentiation. The UCB34 vs. UCB56 post-APC analysis showed markedly reduced expression of KIR2DL1 and KIR3DL1. Another notable finding was that pre-APC UCB34 and UCB56 NK cells and post-APC UCB34 and UCB56 NK cells clustered together, rather than the clustering by original tissue of derivation indicating the influence of APC expansion. Interestingly, several of the most highly differentially expressed genes in UCB34 NK vs. UCB56 NK cells post-APC expansion, such as OSM and LIF, are involved in maintenance of a undifferentiated stem cell phenotype (50, 51). The etiology of the difference in killing ability is likely multifactorial as NK cell cytotoxicity is modulated by diverse signaling pathways, where the net signaling input from both activating and inhibitory receptors determines function (52). Our derivation of UCB34 and UCB56 NK cells from individual donors controls for genetic differences and allows for the comparison of NK cell activity based on developmental origin. However, one possible confounder is that both the UCB56 and UCB34 NK cells were expanded using irradiated aAPCs which provide key activating signals including IL21 and 4-1BB (CD137) stimulation (41). In addition to the same genetic background, we found that the NK cell surface receptor profiles between the UCB34 and UCB56 NK cells are highly similar, primarily differing in CD2, CD16, and KIR expression, all markers that have been associated with NK cell differentiation (47, 53). The fact that KIR^+^ UCB56 cells kill better than KIR^−^ UCB34 cells is similar to reports demonstrating that PB-NK cells that express more KIRs have increased cytotoxic function (54–56). The increased function of KIR^+^ PB-NK cells is thought to be due to the functional maturation associated with NK cell differentiation and licensing. It is unclear if and how these processes control UCB-NK and iPSC-NK cell killing activity. UCB NK cells' cytotoxic activity is reported to be influenced by diverse stimuli including gestational age, mode of delivery, exposure to anesthetic medications, and the presence or absence of infection (57–59).

Given that the major difference we identified between the UCB cell populations was in their KIR expression, we investigated the importance of this difference in KIR expression using the 721.221 lymphoblastoid cell line system. This system allowed us to express HLA C1 (cw3) or C2 (cw4) to otherwise identical cells. Importantly, UCB56 cells from all donors expressed KIR2DL3 and KIR2DL1 while the UCB34 cells from all donors did not express these KIRs. Since the KIR ligands HLA C1 (ligand to KIR2DL3) and C2 (ligand to KIR2DL1) did not significantly alter the difference in killing activity between the UCB56 and UCB34 cells, the canonical HLA recognition role of KIRs does not explain the difference in killing activity in this system. Expression of HLA C1 and HLA C2 would have been expected to decrease the killing ability of the KIR2DL3 and KIR2DL1 expressing UCB56 cells more than it affected the KIR^−^ UCB34 cells if the KIR expression was driving the entire difference in cytotoxicity between the two cell types. Similar cytotoxicity results were obtained against neuroblastoma cell lines which also vary by KIR ligand HLA-C expression although HLA class I expression is generally low in these cells. Thus, in this system KIR expression is associated with enhanced functionality. The inhibitory receptors expressed by the UCB and iPSC-derived NK cells do not seem to interfere with killing of targets expressing high levels of the cognate KIR, rather these cells have increased cytotoxic activity. It is possible that feeder-based expansion drives differentiation at the same time it attenuates inhibitory signaling. Other possible influences include that the effector cells are polyclonal and not all NK cells can be inhibited. Activating KIRs, such as KIR2DS1 in the presence of Cw4, may influence these results as well. However, despite polyclonal expression we do not see an inhibitory effect in UCB56 NK lines that express the relevant KIRs at high frequencies.

NK cells develop their characteristic ability to distinguish between self and non-self through the process of NK cell education, after which they are termed licensed (licensed to kill non-self cells). When an NK cell engages a normal “self” cell, interactions between receptors, such as KIRs, and the matching MHC class I molecule ligand inhibits NK cell lytic function (60, 61). PB-NK cells that lack this licensing step are hypofunctional as the basal level of activity in response to activating receptor signaling corresponds to the number of inhibitory receptors expressed (54, 62). A potential mechanism for this may be that NK cells that express self-specific inhibitory receptors modulate the lysosomal compartment to accumulate dense-core secretory lysosomes containing granzyme B and also uniquely compartmentalize activating and inhibitory receptors on the plasma membrane (63–65). For the donor- derived NK cells in this study, KIR^+^ NK cells represent educated NK cells since donors NK cells were educated by all major KIRs (2DL3, 2DL1, and 3DL1). Our results raise the possibility that KIR-mediated education may be of relevance, although the higher degree of differentiation in the UCB56 lines, which is associated with KIR expression, must also be taken into account. Overall, our results show that increased KIR expression does not impair NK cytotoxicity against a range of target cells that express physiological levels of HLA class I. Since each NK cell population co-expresses many KIRs, it was not possible to delineate the exact role of single KIR expressing NK cells on NK cell education. However, our observation that KIR^+^ NK cells displayed higher functionality is consistent with the notion that self KIR expression boosts functionality through education. As these different NK cell populations are being translated into clinical therapies, it will be important to further elucidate how KIRs contribute and affect the function and specificity of UCB and iPSC-derived NK cells. Our results suggest that KIR^+^ UCB and iPSC-NK cell products are more functional, and their differentiation and expansion appear to attenuate negative signaling supporting broad implementation in an off-the-shelf setting regardless of KIR and HLA genotypes.

## Data Availability Statement

The datasets presented in this study can be found in online repositories. The names of the repository/repositories and accession number(s) can be found below: https://www.ncbi.nlm.nih.gov/geo/, GSE150363 and GSE150806.

## Ethics Statement

The studies involving human participants were reviewed and approved by University of California, San Diego. The patients/participants provided their written informed consent to participate in this study.

## Author Contributions

BG collected and interpreted data and wrote the manuscript. HZ, YW, K-JM, and DK contributed to the conception and design of the study. HZ, YW, NH, AR-C, AB, EA, and HH collected and interpreted data. HZ and YW wrote sections of the manuscript. DK and K-JM reviewed and edited the manuscript. All authors read and approved the submitted manuscript.

## Conflict of Interest

DK: consultant for Fate Therapeutics, has equity, and receives income. The terms of this arrangement have been reviewed and approved by the University of California, San Diego in accordance with its conflict of interest policies. K-JM: consults and receives research support from Fate Therapeutics, has equity and receives income. The terms of this arrangement have been reviewed and approved by the Oslo University Hospital in accordance with its conflict of interest policies. The remaining authors declare that the research was conducted in the absence of any commercial or financial relationships that could be construed as a potential conflict of interest.

## Supplementary Material

Supplementary Figure 1 Schematic of NK cell generation from CD34+ progenitors and iPSCs. **(A)** CD56^+^ UCB NK cells (termed UCB56 NK) and CD34^+^ UCB hematopoietic stem/progenitor cells were isolated from the same donors. The UCB34 cells were then differentiated into NK cells. Representative KIR expression for each NK cell population is indicated. **(B)** NK cells were derived from iPSCs via standard hematopoietic and NK cell differentiation protocols. iPSC-derived NK cells that were derived from different iPSC lines demonstrated significant variability in their KIR expression with distinct KIR positive and KIR negative populations.

Supplementary Figure 2 UCB56 and UCB34 NK cell phenotypes by flow cytometry. NK cell markers by flow cytometry of Donor 1, 2, and 3 UCB34 and UCB56 NK cells (red) compared to isotype controls (blue). Representative panels are shown from *n* = 3 replicates.

Supplementary Figure 3 UCB56 and UCB34 NK cell killing activity against neuroblastoma and myeloid K562 tumors. **(A)** Table of NK cell receptor ligand expression and HLA genotype for neuroblastoma cell lines SK-N-As, IMR32, and NBLS and chronic myeloid leukemia K562 line. **(B)** Cell death and apoptosis by caspase 3,7 activation and 7-AAD staining of SK-N-AS, IMR32, NBLS, and K562 with PBNK cells (blue), UCB56 NK cells (purple), and UCB34 NK cells (orange) after 4-h co-culture at effector:target ratios from 0.3:1 up to 5:1. Representative panels are shown from *n* = 3 replicates. All statistical analysis is of the comparisons between UCB56 and UCB34 NK cells. **(C)** Tumor cells alone (red) and tumor cell killing by PB-NK (blue), UCB56 (purple), and UCB34 NK cells (orange) measured by Incucyte live-imaging system over 24 h. Experiments were completed in triplicate.

Supplementary Figure 4 Gene expression analysis of NK cell cytotoxicity pathway genes by qRT-PCR of UCB NK, PB NK, and iPSC NK cells. The levels of mRNA for the indicated genes were assayed by qRT-PCR. Bar graph depicts means ± SD. Comparisons by fold change between PB NK and iPSC NK cells are indicated in blue, and comparisons by fold change between UCB NK and iPSC NK cells are indicated in orange. Data are representative of two experiments.

Supplementary Table 1 List of antibodies used in mass cytometry experiments.

Supplementary Table 2 List of NK Cell KIR Genotypes and HLA Haplotypes. For HLA typing molecular (Mol) and serological (Sero) typing information is included.
