# Supplementary figures and images for: Umbilical Cord Blood and iPSC-Derived Natural Killer Cells Demonstrate Key Differences in Cytotoxic Activity and KIR Profiles
Source: Front Immunol. 2020 Oct 15;11:561553. doi: 10.3389/fimmu.2020.561553 (PMC7593774; doi:10.3389/fimmu.2020.561553)

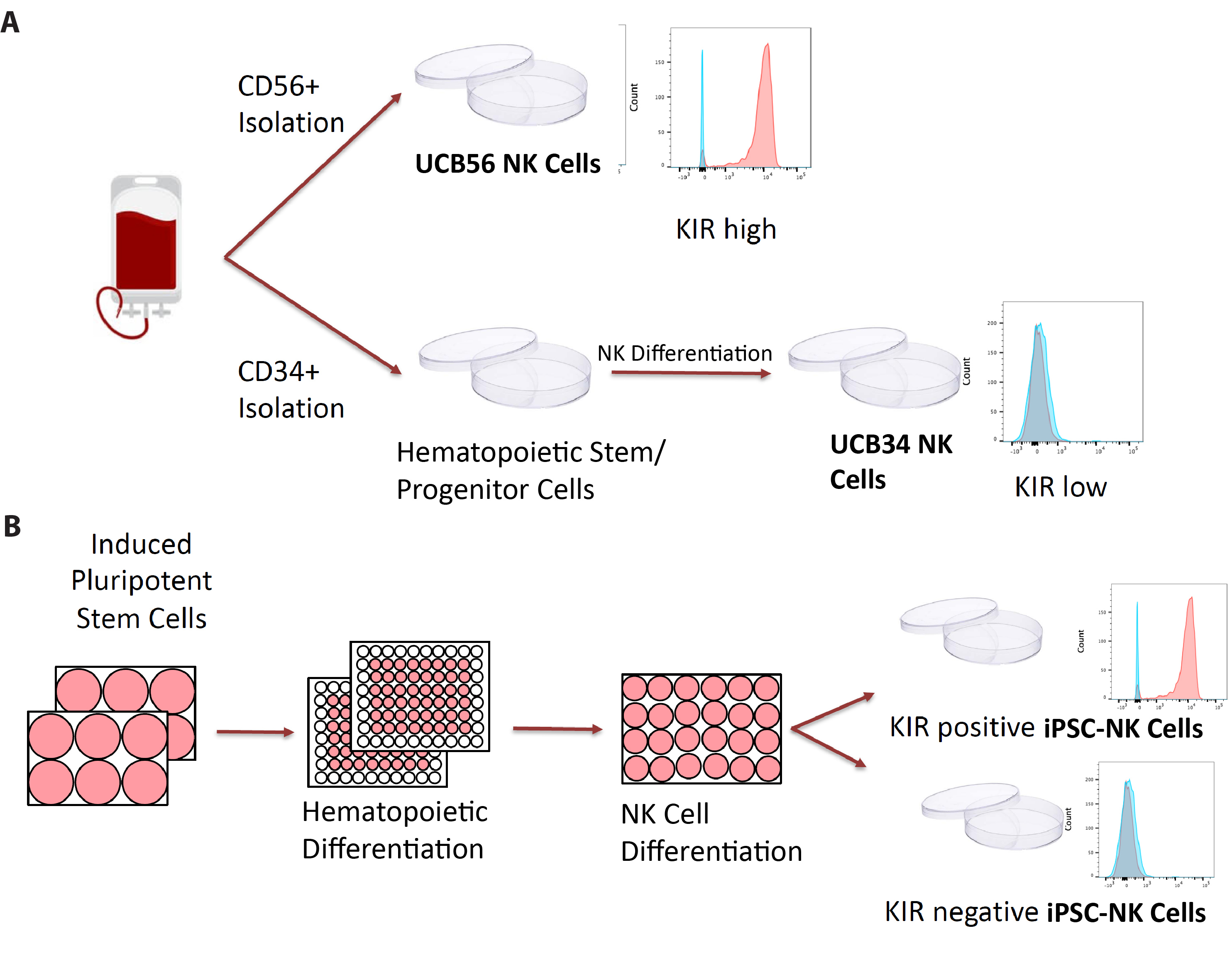

Supplement: Supplementary Figure 1 — Schematic of NK cell generation from CD34+ progenitors and iPSCs. (A) CD56+ UCB NK cells (termed UCB56 NK) and CD34+ UCB hematopoietic stem/progenitor cells were isolated from the same donors. The UCB34 cells were then differentiated into NK cells. Representative KIR expression for each NK cell population is indicated. (B) NK cells were derived from iPSCs via standard hematopoietic and NK cell differentiation protocols. iPSC-derived NK cells that were derived from different iPSC lines demonstrated significant variability in their KIR expression with distinct KIR positive and KIR negative populations. [file Image_1.JPEG]

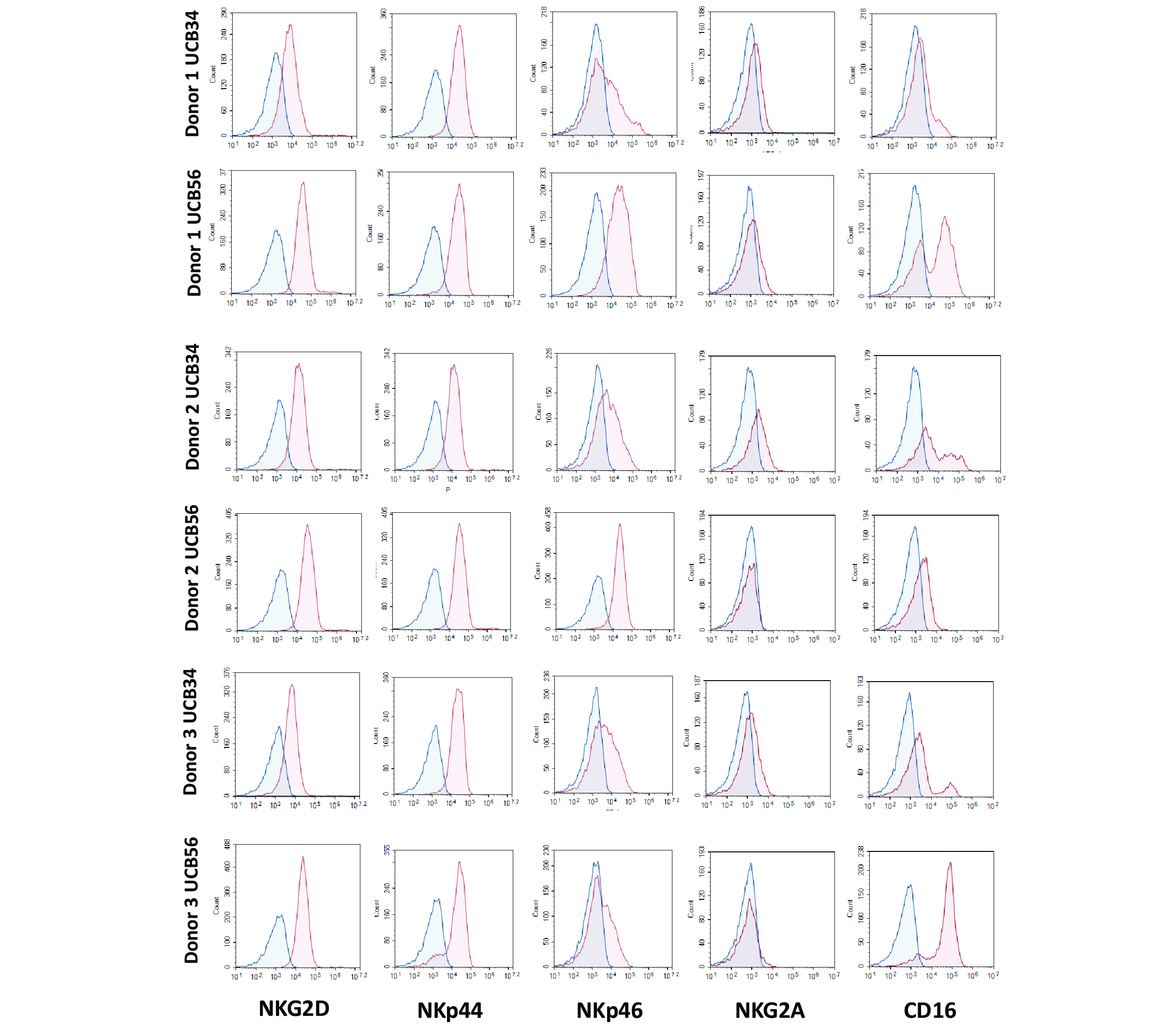

Supplement: Supplementary Figure 2 — UCB56 and UCB34 NK cell phenotypes by flow cytometry. NK cell markers by flow cytometry of Donor 1, 2, and 3 UCB34 and UCB56 NK cells (red) compared to isotype controls (blue). Representative panels are shown from n = 3 replicates. [file Image_2.JPEG]

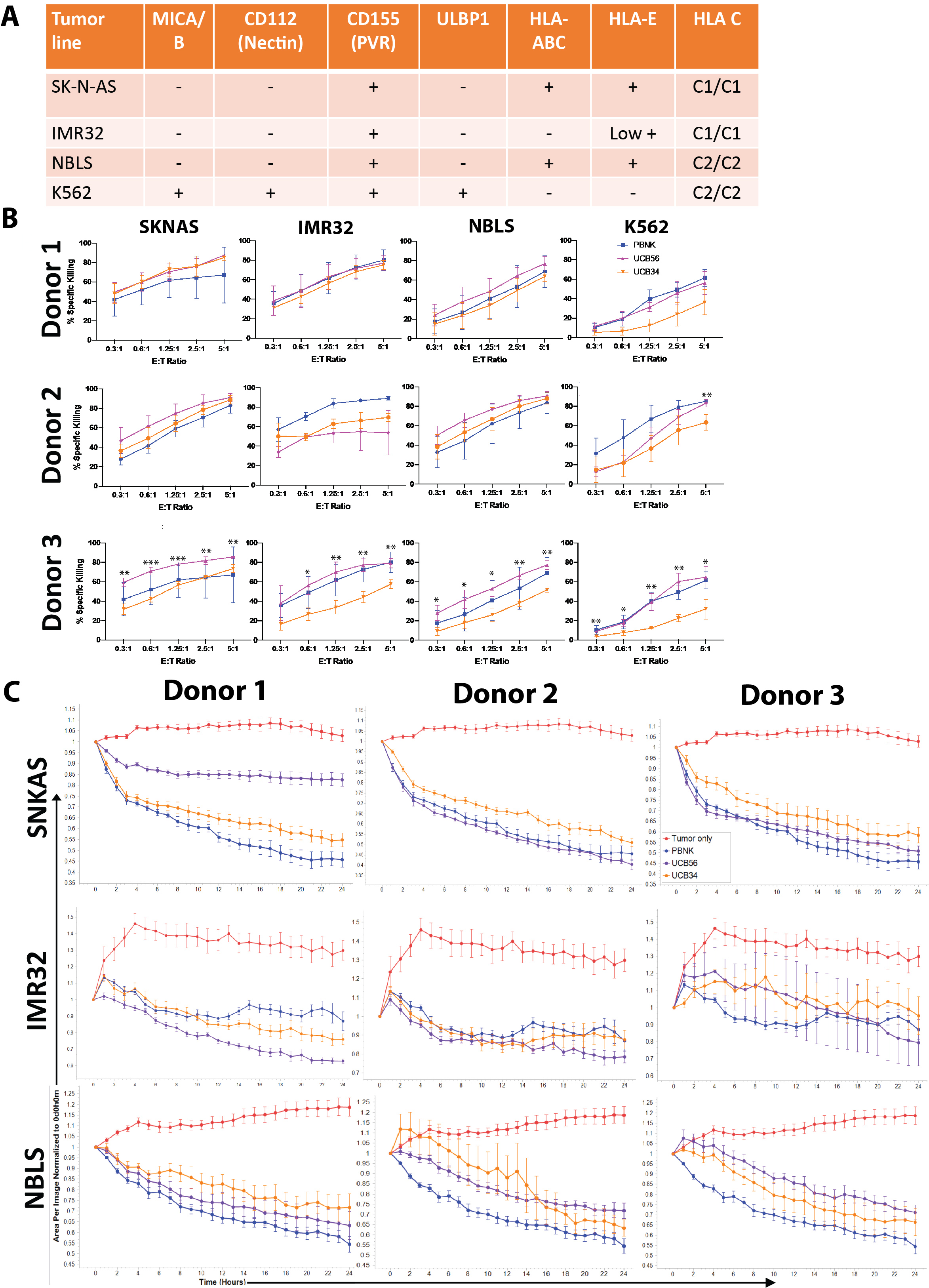

Supplement: Supplementary Figure 3 — UCB56 and UCB34 NK cell killing activity against neuroblastoma and myeloid K562 tumors. (A) Table of NK cell receptor ligand expression and HLA genotype for neuroblastoma cell lines SK-N-As, IMR32, and NBLS and chronic myeloid leukemia K562 line. (B) Cell death and apoptosis by caspase 3,7 activation and 7-AAD staining of SK-N-AS, IMR32, NBLS, and K562 with PBNK cells (blue), UCB56 NK cells (purple), and UCB34 NK cells (orange) after 4-h co-culture at effector:target ratios from 0.3:1 up to 5:1. Representative panels are shown from n = 3 replicates. All statistical analysis is of the comparisons between UCB56 and UCB34 NK cells. (C) Tumor cells alone (red) and tumor cell killing by PB-NK (blue), UCB56 (purple), and UCB34 NK cells (orange) measured by Incucyte live-imaging system over 24 h. Experiments were completed in triplicate. [file Image_3.JPEG]

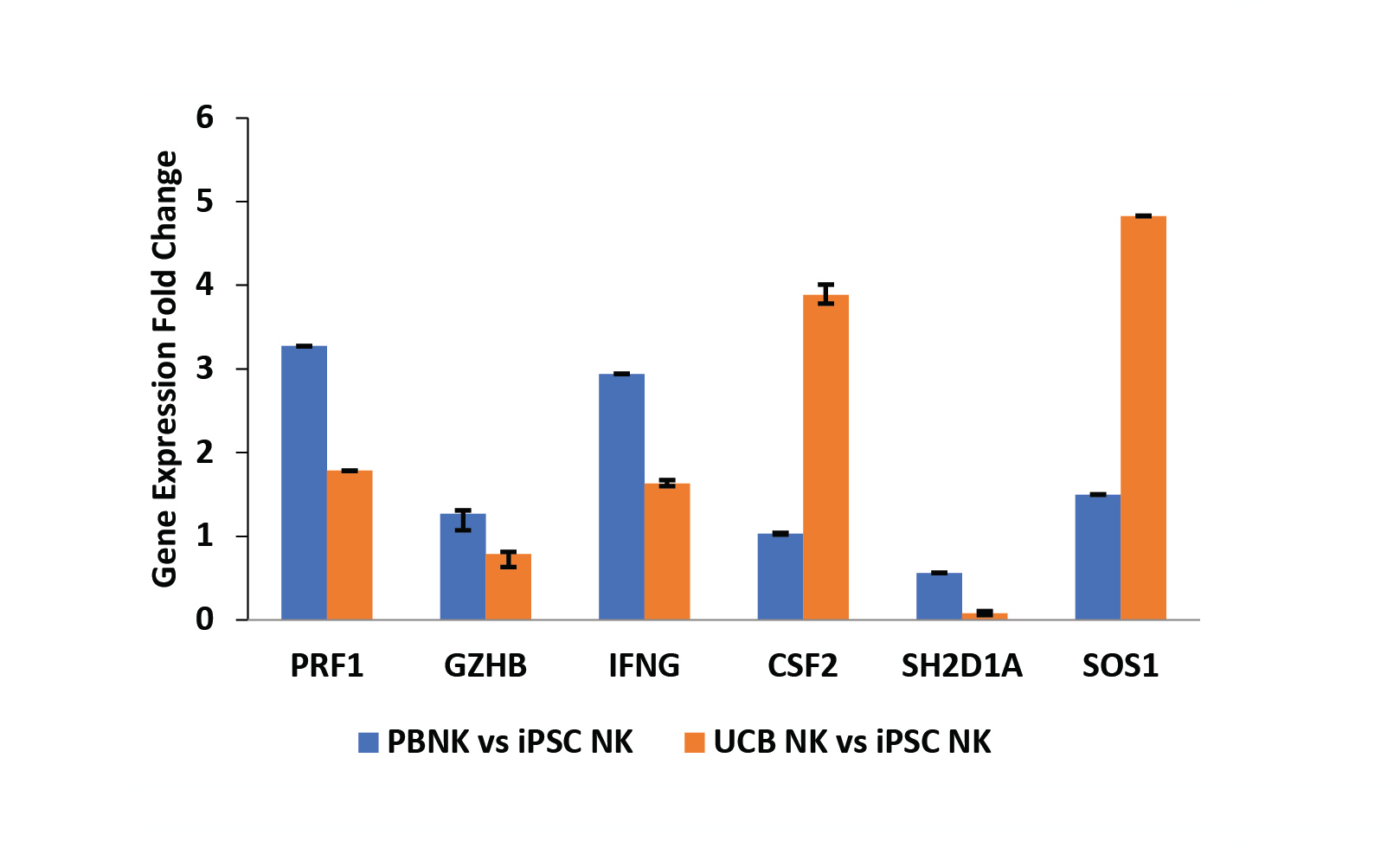

Supplement: Supplementary Figure 4 — Gene expression analysis of NK cell cytotoxicity pathway genes by qRT-PCR of UCB NK, PB NK, and iPSC NK cells. The levels of mRNA for the indicated genes were assayed by qRT-PCR. Bar graph depicts means ± SD. Comparisons by fold change between PB NK and iPSC NK cells are indicated in blue, and comparisons by fold change between UCB NK and iPSC NK cells are indicated in orange. Data are representative of two experiments. [file Image_4.JPEG]
