# Supplementary material for: Umbilical Cord Blood and iPSC-Derived Natural Killer Cells Demonstrate Key Differences in Cytotoxic Activity and KIR Profiles
Source: Front Immunol. 2020 Oct 15;11:561553. doi: 10.3389/fimmu.2020.561553 (PMC7593774; doi:10.3389/fimmu.2020.561553)
Supplement: Supplementary Table 1 — List of antibodies used in mass cytometry experiments. [file Table_1.PDF]

Supplemental Table 1. List of antibodies used in mass cytometry experiments.

Tag	Marker	Clone	Isotype	Vendor	Catalog number
89Y	CD45	HI30	IgG1	Fluidigm	3089003B
Qdot655	CD19	SJ25-C1	Mouse IgG1 kappa	Invitrogen	Q10179
141Pr	KIR2DS4	JJC11.6	Mouse IgG1 kappa	Miltenyi	130-092-679
142Nd	CD57	HCD57	Other	Fluidigm	3142007B
143Nd	2B4	REA112	Recomb. human IgG1	Miltenyi	130-124-523
144Nd	CD38	REA572	Recomb. human IgG1	Miltenyi	130-122-307
PE	CD32	REA997	Recomb. human IgG1	Miltenyi	130-116-599
145Nd	PE	PE001	Mouse IgG1	Fluidigm	3145006B
146Nd	CD8	RPA-T8	Mouse IgG1	Fluidigm	3146001B
147Sm	KIR3DL2	DX31	Unknown	Mabtech	Custom
148Nd	CD14	RMO52	Mouse IgG2a	Fluidigm	3148010B
149Sm	Syk	4D10.2	Mouse IgG2a	Fluidigm	3149020B
150Nd	CD34	AC136	Mouse IgG2a	Miltenyi	130-108-040
151Eu	TIGIT	MBSA43	Mouse IgG1 kappa	Invitrogen	16-9500-82
152Sm	Siglec-7	194211	Mouse IgG1	R&D Systems	MAB11381
153Eu	TIM-3	F38-2E2	Unknown	Miltenyi	Custom
154Sm	NKG2C	REA205	Recomb. human IgG1	Miltenyi	130-122-278
155Gd	KIR2DL1/S1	11PB6	Mouse IgG1 kappa	Miltenyi	130-092-682
156Gd	LILRB1	GHI/75	IgG2b	Fluidigm	3156020B
158Gd	KIR2DL1	REA284	Recomb. human IgG1	Miltenyi	130-122-279
159Tb	CD2	RPA-2.10	Mouse IgG1 kappa	eBioscience	14-0029-82
161Dy	Ki-67	B56	Mouse IgG1	Fluidigm	3161007B
162Dy	CD27	L128	Mouse IgG1	Fluidigm	3162009B

163Dy	KIR2DL3	REA147	Recomb. human IgG1	Miltenyi	130-122-280
164Dy	CD161	HP-3G10	Mouse IgG1	Fluidigm	3164009B
165Ho	KSP37	TDA3	Mouse IgG1 kappa	Biolegend	346603
166Er	NKG2D	ON72	IgG1	Fluidigm	3166016B
167Er	KIR3DL1	DX9	IgG1	Fluidigm	3167013B
168Er	NKp30	AF29-4D12	Mouse IgG1 kappa	Miltenyi	130-092-554
169Tm	NKG2A	Z199	IgG2b	Fluidigm	3169013B
170Er	CD3	UCHT1	Mouse IgG1	Fluidigm	3170001B
171Yb	DNAM-1	DX11	IgG1	Fluidigm	3171013B
172Yb	KIR2DL2/L3/S2	GL183	Mouse IgG1	Beckman Coulter	IM1846
173Yb	Granzyme B	GB11	Mouse IgG1	Fluidigm	3173006B
174Yb	CD94	HP-3D9	IgG2a	Fluidigm	3174015B
175Lu	Perforin	B-D48	Mouse IgG1	Fluidigm	3175004B
176Yb	CD56	NCAM16.2	Mouse IgG2b	Fluidigm	3176008B
209Bi	CD16	3G8	IgG1	Fluidigm	3209002B
