# Supplementary material for: Umbilical Cord Blood and iPSC-Derived Natural Killer Cells Demonstrate Key Differences in Cytotoxic Activity and KIR Profiles
Source: Front Immunol. 2020 Oct 15;11:561553. doi: 10.3389/fimmu.2020.561553 (PMC7593774; doi:10.3389/fimmu.2020.561553)
Supplement: Supplementary Table 2 — List of NK Cell KIR Genotypes and HLA Haplotypes. For HLA typing molecular (Mol) and serological (Sero) typing information is included. [file Table_2.PDF]

Supplemental Table 2. List of NK Cell KIR Genotypes and HLA Haplotypes. For HLA typing molecular (Mol) and serological (Sero) typing information is included.

KIR Genotypes	Cen A	Cen B	Tel A	Tel B	2DS1	2DS2	2DS3	2DS4	2DS5	3DS1	2DL1	2DL2	2DL3	2DL4	2DL5	2DP1	3DL1	3DL2	3DL3	3DP1
UCB Donor 1	+	+	+	+	+	+	-	+	+	+	+	+	+	+	+	+	+	+	+	+
UCB Donor 2	+	+	-	+	+	-	+	-	+	+	+	+	+	+	+	+	-	+	+	+
UCB Donor 3	+	+	+	-	-	-	+	+	-	-	+	+	+	+	+	+	+	+	+	+
KIRNeg iPSC-NK	-	+	-	+	+	+	+	-	+	+	+	+	-	+	+	+	-	+	+	+
KIRPos iPSC-NK	+	+	+	+	+	+	-	+	+	+	+	+	+	+	+	+	+	+	+	+

HLA Haplotypes	A		A		B (Bw)		B (Bw)		C		C	
	Mol	Sero	Mol	Sero	Mol	Sero	Mol	Sero	Mol	Sero	Mol	Sero
UCB Donor 1	*11	A11	*11	A11	*52	B52 (4)	*35	B35 (6)	*04	4 (C2)	*12	12 (C1)
UCB Donor 2	*11	A11	*33	A33	*44	B44 (4)	*14:02	B65 (6)	*05	5 (C2)	*08	8 (C1)
UCB Donor 3	*26	A26	*33	A33	*58	B58 (4)	*56	B56 (6)	*03:02	10 (C1)	*07	7 (C1)
KIRNeg iPSC-NK	*11	A11	*68	A68	*52	B52 (4)	*57	B57 (4)	*06	6 (C2)	*12	12 (C1)
KIRPos iPSC-NK	*23	A23	*25	A25	*07	B7 (6)	*08	B8 (6)	*04	4 (C2)	*12	12 (C1)
